# Supplementary material for: Tristetraprolin inhibits macrophage IL-27-induced activation of antitumour cytotoxic T cell responses
Source: Nat Commun. 2017 Oct 11;8:867. doi: 10.1038/s41467-017-00892-y (PMC5636828; doi:10.1038/s41467-017-00892-y)
Supplement: Supplementary file 1 — Supplementary Information [file 41467_2017_892_MOESM1_ESM.pdf]

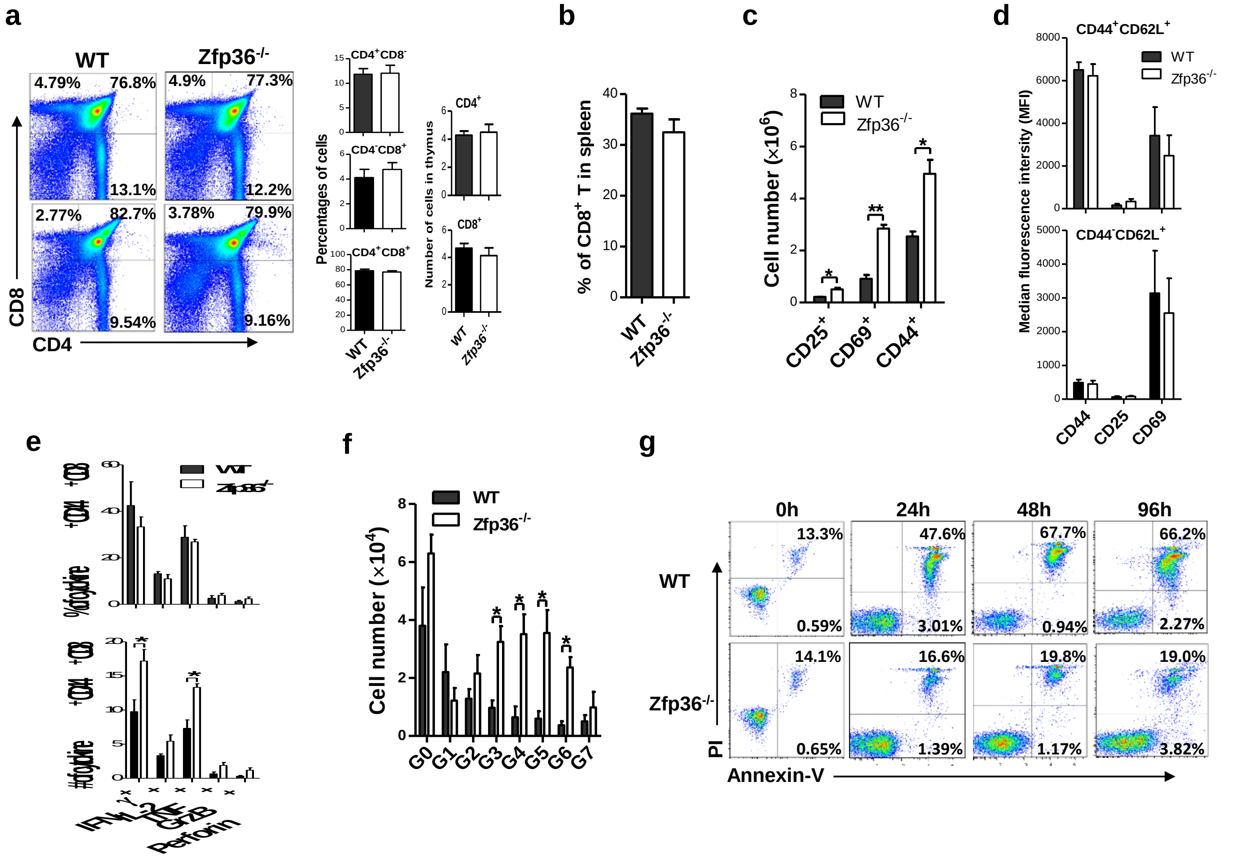

Supplementary Fig. 1. TTP deficiency promotes proliferation, survival and activation of CD8$^+$ T cells. (**a**) Thymocytes of female *Zfp36*$^{-/-}$ and WT littermates aged 6-8 weeks old were stained by anti-CD4 and CD8 antibodies, followed by detection by FACS gated on total thymocytes. The percentages of CD4$^+$, CD8$^+$ and CD4$^+$CD8$^+$ T cells as well as the numbers of CD4$^+$ and CD8$^+$ T cells in thymus were summarized as means ± s.d. from three pairs of mice. (**b**) Splenocytes from the above mice were stained by anti-CD3 and CD8 antibodies. The percentages of CD8$^+$ T cells in spleen were determined with FACS by gating on CD3$^+$ cells (n=6). (**c**) Splenocytes were isolated from female *Zfp36*$^{-/-}$ mice and WT littermates at 6 weeks old, and stained with antibodies against CD3, CD8, CD44, CD25, CD69 and CD62L. The CD25$^+$, CD69$^+$, CD44$^+$ and CD62L$^+$ CD8 T cells were detected with FACS gated on CD3$^+$CD8$^+$ double positive cells. The absolute numbers were calculated from three pairs of mice and

analyzed with unpaired student's *t* test. (**d**) The levels of CD44, CD25 and CD69 as in (c) were detected by FACS in $CD3^+CD8^+$ double positive cells. The median fluorescence intensities of CD44, CD25 and CD69 in $CD44^+CD62L^+$ and $CD44^-CD62L^+$ cells were calculated and summarized as means ± s.d. with three mice in each group. (**e**) The splenocytes as in (c) were stimulated by PMA and Ionomycin in the presence of GolGistop for 4 h, then intracellular IFN-γ, IL-2, TNF, Granzyme B and Perforin positive cells were detected with their respective antibodies, gated on CD44 in $CD3^+$ and $CD8^+$ double positive cells. Cytokine production was detected in the $CD44^+$ cells. Quantitative data represent means ± s.d. of $cytokine^+CD8^+$ T cells from three pair of mice. (**f**) Naïve CD8 T cells isolated from spleens of WT and *Zfp36*$^{-/-}$ mice were labeled by CFSE and stimulated by plate-coated α-CD3/CD28 Abs (1 μg/ml) for 72 h. Then proliferative generations were analyzed based on CFSE levels. Quantitative data represent means ± s.d. from three independent experiments. G represents generation. (**g**) Naïve CD8 T cells of WT and *Zfp36*$^{-/-}$ mice were stimulated by plate-coated α-CD3/CD28 Abs (1 μg/ml) for different times. PI and Annexin-V expression were detected with FACS by gating on $CD3^+CD8^+$ cells. Data represent one of three experiments with similar results. *: $p<0.05$; **: $p<0.01$; ***: $p<0.001$ between WT and *Zfp36*$^{-/-}$ groups.

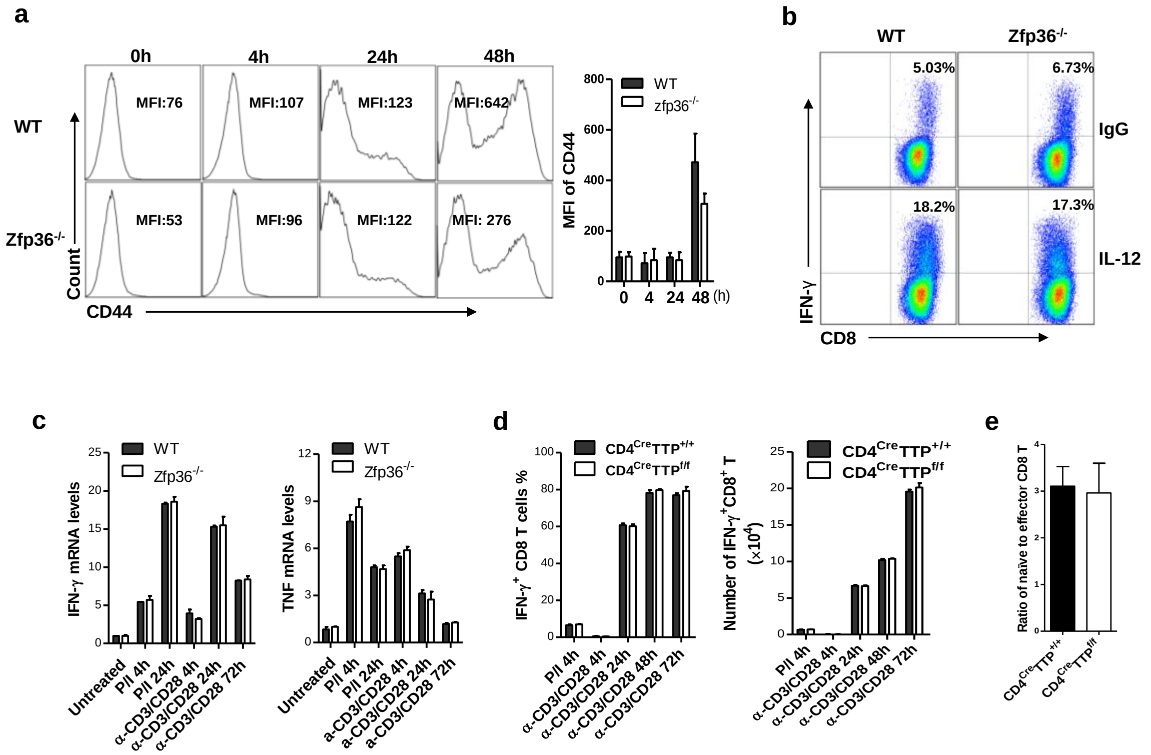

Supplementary Fig. 2. TTP has no intrinsic effects on IFN-γ production by CD8⁺ T cells. (**a**) Naïve CD8$^+$ T cells isolated from WT and *Zfp36*$^{-/-}$ mice were stimulated with plate-coated α-CD3 and soluble α-CD28 Abs (1 μg/ml), then CD44 was detected at different times. Median fluorescence intensity (MFI) was shown as histogram and summarized from three experiments (means ± s.d.). (**b**) Naïve CD8 T cells from WT and *Zfp36*$^{-/-}$ mice were stimulated by plate-coated anti-CD3/CD28 Abs (1 μg/ml) in the presence of IgG or recombinant mouse IL-12 (1 ng/ml) for 3 day. Then IFN-γ production was detected with FACS gated on CD3$^+$CD8$^+$ cells. Date shown represent one of two experiments with similar results. (**c**) Naïve CD8 T cells were stimulated by PMA (50 ng/ml) and Ionomycin (1 μg/ml) or by plate-coated anti-CD3/CD28 Abs (1 μg/ml) for different times as indicated. Total RNA was extracted. IFN-γ and TNF mRNA levels were detected by qRT-PCR. The qRT-PCR data were normalized relative to GAPDH mRNA levels and further normalized to the results from untreated group. Results shown are

means ± s.e.m of three independent experiments. (**d**) Splenocytes of $CD4^{Cre}TTP^{+/+}$and $CD4^{Cre}TTP^{f/f}$ mice were stimulated by PMA and Ionomycin in the presence of GolGistop for 4h or by soluble anti-CD3/CD28 Abs (1 μg/ml) for different time points as indicated. IFN-γ production was detected with FACS by gating on $CD3^{+}CD8^{+}$ cells. The percentages and numbers of IFN-γ^{+}CD8 T cells in $CD8^{+}$ population were summarized from three independent experiments (means ± s.d.). (e) Splenocytes of $CD4^{Cre}TTP^{+/+}$and $CD4^{Cre}TTP^{f/f}$ mice were stained for CD3, CD8 and CD44. $CD44^{+}$ and $CD44^{-}$ cells were counted by gating on $CD3^{+}CD8^{+}$ population. The ratio of naïve to effector cells were calculated as $CD44^{-}$ cells vs. $CD44^{+}$ cells. Data shown are means ± s.d. of four experiments.

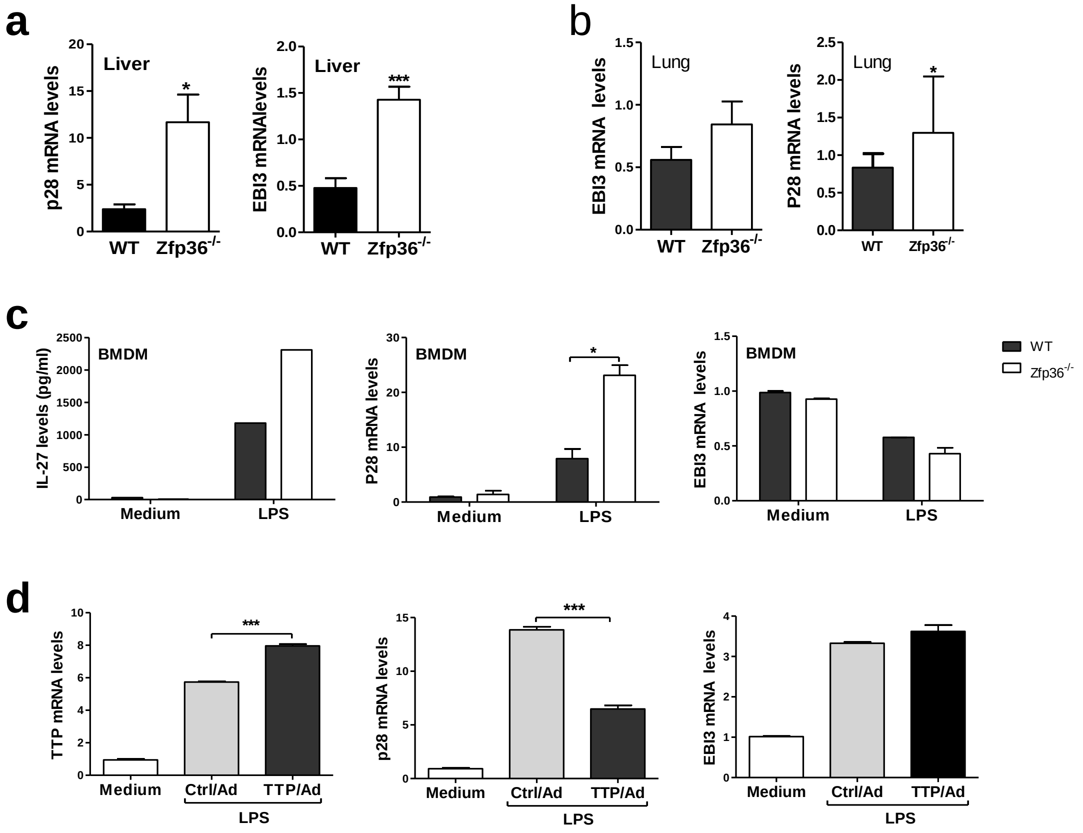

Supplementary Fig. 3. IL-27 expression is increased in *Zfp36*$^{-/-}$ macrophages. (**a,b**) p28 and EBI3 mRNA in liver and lung of WT and *Zfp36*$^{-/-}$ mice were detected by qRT-PCR and normalized relative to GAPDH mRNA levels and further normalized to the results from WT mice. Results shown are means ± s.e.m of five mice in each group and analyzed with unpaired Students' *t* test. (**c**) BMDM derived bone marrow cells of WT and *Zfp36*$^{-/-}$ mice were stimulated by LPS for 4 h. Then, p28 and EBI3 mRNA, and IL-27 protein were detected by qRT-PCR and ELISA, respectively (n=3). (**d**) J774 cells were infected by control/adenovirus (Ctrl/Ad) or TTP/adenovirus (TTP/Ad) for 48 h, and then stimulated by LPS for 4 h. The mRNAs of TTP, p28 and EBI3 were detected by qRT-PCR. Data shown are means ± s.e.m. from three independent experiments and analyzed with unpaired Students'*t* test. *: p<0.05; **: p<0.01; ***: p<0.001.

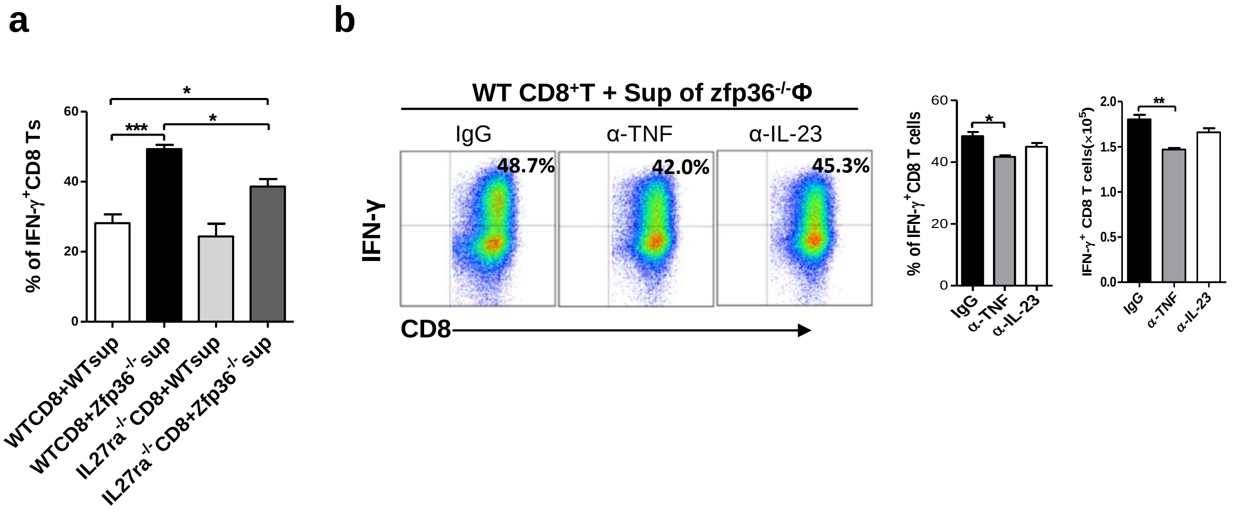

Supplementary Fig.4. IL-27 derived from *Zfp36*$^{-/-}$ macrophages enhances IFN-γ production by CD8 T cells. (**a**) WT and *Zfp36*$^{-/-}$ peritoneal macrophages were stimulated by LPS (1 μg/ml) for 24 h, and then supernatants were collected. Purified naïve WT and *IL27ra*$^{-/-}$ CD8$^+$ T cells were cultured with the supernatants (1:1) in the presence of plate-coated α-CD3/CD28 Abs (1 μg/ml) for 3 days. The percentages of IFN-γ$^+$CD8$^+$T cells were detected by FACS and shown as means ± s.d. from three independent experiments. (**b**) Supernatants of the *Zfp36*$^{-/-}$ peritoneal macrophages were respectively pre-treated with TNF and IL-23 neutralizing antibodies (10 μg/ml) for 30 min, and then cultured with WT naïve CD8 T cells (supernatant vs. medium=1:1) in the presence of plate-coated α-CD3/CD28 Abs (1 μg/ml) for 3 days. IFN-γ$^+$CD8$^+$T cells were detected by FCM. Quantitative data shown are means ± s.d. from three independent experiments. One-way ANOVA with Turkey was used as in (a & b), *: p<0.05; **: p<0.01; ***: p<0.001 between groups.

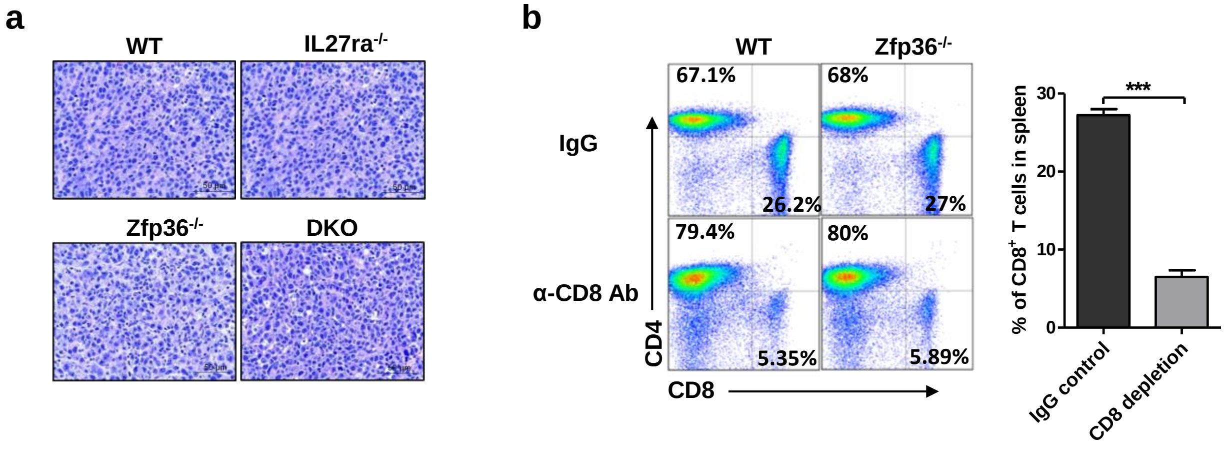

Supplementary Fig. 5. TTP-mediated tumor progression is dependent on CD8 T cells. (**a**) 0.5×10^6 EO771 cells were inoculated into mammary gland pads of WT, *IL27ra*$^{-/-}$, *Zfp36*$^{-/-}$, and DKO mice. Twenty-four days after tumor cell inoculation, mice were sacrificed and tumors were analyzed with HE staining (magnification 100×). (**b**) Splenocytes purified from the tumor-bearing mice receiving CD8 depletion antibody as in Fig. 6j were stained with antibodies against CD3, CD4 and CD8, then detected by FACS. The percentages of CD4$^+$ and CD8$^+$ T cells were determined by gating on CD3$^+$ cells. Bar shows means ± s.d. with four samples in each group (unpaired Student's *t* test, ***: $p<0.001$).

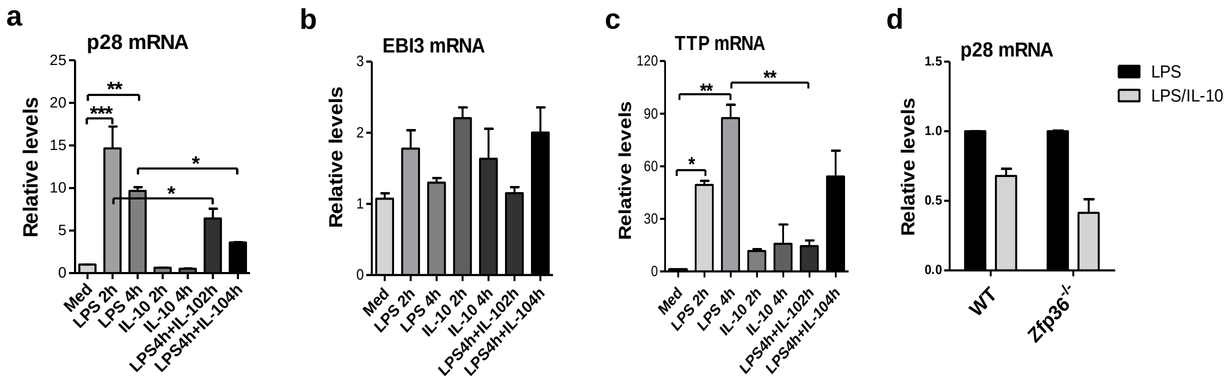

Supplementary Fig. 6. IL-10 inhibits IL-27 production independent of TTP. (**a-c**) BMDMs of WT mice were stimulated by LPS (1 μg/ml), recombinant mouse IL-10 (10 ng/ml), or combination of both for 2 and 4 hrs, respectively. The mRNA levels of IL-27 p28 (**a**), EBI3 (**b**) and TTP (**c**) were detected by qRT-PCR. The qRT-PCR data were normalized relative to GAPDH mRNA levels and further normalized to the results from untreated group (Med). Results shown are means ± s.e.m of three independent experiments and analyzed with one-way ANOVA (Nonparametric) with Tukey (compare all pairs of columns). (**d**) BMDMs of WT and *Zfp36*$^{-/-}$ mice were stimulated by LPS (1 μg/ml) with or without IL-10 (40 ng/ml) for 2h. Then p28 mRNA was detected by qRT-PCR. Results shown are means ± s.d. of three independent experiments and analyzed with unpaired two-tailed Student's *t* test. *: p<0.05; **: p<0.01; ***: p<0.001 between indicated groups.

Supplementary Table l. **Antibody list**

Name	Company	Catalog number	Clone number
Anti-Mouse CD3 APC-780	eBioscience	47-0032-82	17A2
Anti-Mouse CD3 Percp-cy5.5	Biolegend	100217	17A2
Anti-Mouse CD4 percp	BD Biosciences	553052	RM4-5
Anti-Mouse CD8 FITC	eBioscience	11-0081-82	53-6.7
Anti-Mouse CD8 PE	BD Biosciences	552877	53-6.7
Anti-Mouse CD8 APC-780	eBioscience	47-0032-82	53-6.7
Anti-Mouse IFN-γ APC	BD Biosciences	554413	XMG1.2
Anti-Mouse IL-2 FITC	eBioscience	11-7021-41	JES6-5H4
Anti-mouse TNF-α FITC	eBioscience	11-7321-81	MP6-XT22
Anti-mouse Granzyme B PE-CY7	eBioscience	25-5831-82	GzA-3G8.5
Anti-mouse Perforin APC	eBioscience	17-9392-80	eBioOMAK-D
Anti-mouse CD25 PE	eBioscience	12-0251-81	PC61.5
Anti-mouse CD69 Percy-cy5.5	eBioscience	45-0691-80	H1.2F3
Anti-Human/Mouse CD44 FITC	eBioscience	11-0441-81	IM7
Anti-Mouse CD62L	BD Biosciences	560516	MEL-14
Anti-mouse Ki-67 PE	Biolegend	652404	16A8
Anti-Mouse CD8a Purified	eBioscience	14-0081-82	53-6.7
Anti-Mouse CD45 PE-cy7	eBioscience	25-0451	30-F11
Anti-TTP (N-terminal)	Sigma	T5327	polyclonal
Anti-mouse IL-12/IL-23 p40	Biolegend	505304	C17.8
Anti-mouse IL-23 p19	Biolegend	513805	MMp19B2
Anti-mouse TNF-α	eBioscience	14-7423-85	TN3-19.12
Anti-mouse CD8α	Bio X cell	BP0004-1	53-6.7
